# Advancements in Nanogels for Enhanced Ocular Drug Delivery: Cutting-Edge Strategies to Overcome Eye Barriers

**DOI:** 10.3390/gels9090718

**Published:** 2023-09-04

**Authors:** Hyeonah Lee, Hyeran Noh

**Affiliations:** Department of Optometry, Seoul National University of Science and Technology, Gongnung-ro 232, Nowon-gu, Seoul 01811, Republic of Korea; leeha0653@seoultech.ac.kr

**Keywords:** nanomedicine, nanogel, ocular therapy, targeting modulators, DNA

## Abstract

Nanomedicine in gel or particle formation holds considerable potential for enhancing passive and active targeting within ocular drug delivery systems. The complex barriers of the eye, exemplified by the intricate network of closely connected tissue structures, pose significant challenges for drug administration. Leveraging the capability of engineered nanomedicine offers a promising approach to enhance drug penetration, particularly through active targeting agents such as protein peptides and aptamers, which facilitate targeted release and heightened bioavailability. Simultaneously, DNA carriers have emerged as a cutting-edge class of active-targeting structures, connecting active targeting agents and illustrating their potential in ocular drug delivery applications. This review aims to consolidate recent findings regarding the optimization of various nanoparticles, i.e., hydrogel-based systems, incorporating both passive and active targeting agents for ocular drug delivery, thereby identifying novel mechanisms and strategies. Furthermore, the review delves into the potential application of DNA nanostructures, exploring their role in the development of targeted drug delivery approaches within the field of ocular therapy.

## 1. Introduction

Ocular drug delivery presents significant challenges due to the intricate anatomy and physiology of the eye, requiring innovative strategies to effectively treat various ophthalmic conditions. The eye, a highly specialized organ responsible for vision, is protected by numerous barriers [1,2]. The barriers—such as the cornea, conjunctiva, sclera, and blood–retinal barrier (BRB)—are designed to prevent harmful substances from entering while permitting essential nutrients and oxygen to pass through. Traditional ocular drug delivery methods, which mainly involve eye drops and ointments, face limitations in terms of drug bioavailability and efficacy due to low residence time, poor penetration, and potential systemic side effects [3]. Furthermore, these methods often lack precision in delivering therapeutic agents to specific regions within the eye, leading to suboptimal treatment outcomes [4,5]. Hence, there is an increasing need for the development of innovative drug delivery systems that can overcome these limitations, enhance treatment outcomes, and ultimately improve the quality of life for patients with ophthalmic diseases.

In recent years, nanotechnology has emerged as a promising approach to address ocular drug delivery challenges, with hydrogel-based systems being an example of the advancements made in this field [6,7,8]. Nanoparticles, which range from 1 to 100 nanometers in size, are considerably smaller than the cells composing ocular barriers. Owing to their size advantage, nanoparticles can potentially penetrate the cornea and reach the eye’s deeper layers, such as the retina and choroid, delivering drugs directly to the target site [9]. These drug delivery systems often exhibit improved pharmacokinetic profiles and can be tailored to release the therapeutic agent in a controlled manner, enhancing both safety and efficacy. For example, by incorporating nanoparticles within the hydrogel matrix, it is possible to achieve enhanced drug retention time, better protection from premature degradation, and controlled release of the therapeutic agent, thus overcoming various challenges associated with ocular drug delivery [10]. However, the development of further effective ocular drug delivery systems necessitates a comprehensive understanding of the ocular barriers and factors influencing drug permeation. To improve interaction with biological barriers, researchers have investigated several strategies for modulating the physicochemical properties of nanoparticles, including surface charge, particle size, and shape, which can influence drug delivery efficiency [11,12,13]. Additionally, incorporating specific ligands that can bind to receptors on target cells or tissues has shown promise for actively targeting drug delivery systems to the desired site of action [14,15]. Improving these barriers necessitates strategies that amplify permeability and retention by passively targeting specific sites, modulating the surface charge, and adjusting the physical and chemical properties of nanocarriers. Precise and sensitive targeted drug delivery can be achieved using targeting molecules such as proteins and aptamers capable of identifying targets, yielding promising research results [16,17]. In light of this trend, DNA emerges as a new therapeutic method, exhibiting high biocompatibility, design flexibility, and compatibility, fulfilling the requirements for therapeutic molecules acting on the eye’s precise organs. Consequently, using DNA for targeted ocular drug delivery constitutes a promising therapeutic approach. Researchers are investigating varied types of DNA nanostructures and external-responsive DNA carriers, including DNA nanogel, to develop sophisticated ocular drug delivery systems with improved specificity and therapeutic efficacy [18,19,20].

This review discusses ocular barriers and challenges associated with ocular drug delivery. Additionally, recent advances in nanotechnology-based ocular drug delivery systems and strategies for overcoming ocular barriers are examined. The limitations of current research, standardization needs, and potential future research developments are also addressed. In conclusion, this review aims to provide a comprehensive understanding of the challenges, advancements, and future directions in ocular drug delivery, ultimately contributing to the ongoing progress and innovation in this research area.

## 2. Barriers to Drug Administration for Ocular Diseases

**Figure 1:**
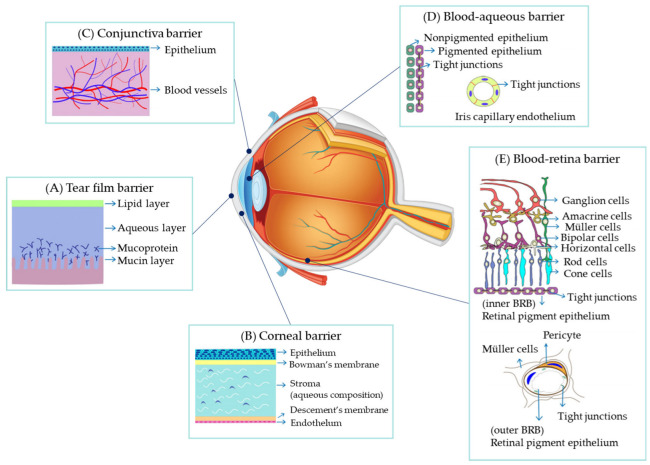
Structures of ocular barriers to drug delivery. Drug delivery encounters several barriers, including the tear film, corneal, conjunctival, blood–aqueous, and blood–retinal barriers. These barriers function to protect the eye from foreign substances (tear film), limit drug absorption (corneal and conjunctival), and restrict drug passage from the blood to the aqueous humor or retina (blood–aqueous and blood–retinal). (**A**) The tear film barrier comprises lipid, aqueous, and mucous layers functioning as a protective shield, preventing foreign substances from reaching the cornea and conjunctiva. (**B**) The corneal barrier, consisting primarily of tightly connected epithelial cells, soluble stroma, and a single endothelial cell layer, hinders drug absorption from tear fluid into the anterior chamber following topical application. (**C**) The conjunctival barrier, a mucous membrane formed by the conjunctival epithelium and the underlying vascular connective tissue, provides a greater extensive absorption area than that of the cornea. However, drugs are easily eliminated through capillaries and enter systemic circulation. (**D**) The anterior chamber’s blood-aqueous barrier, formed by the iris capillary endothelium and the ciliary body’s non-pigmented epithelium, contains tight junctions that obstruct drug passage into the aqueous humor from the blood. (**E**) The blood-retina barrier, situated in the eye’s posterior segment, is created by the retinal pigment epithelium and the retinal blood vessels endothelium. Tight junctions within this barrier also limit the entry of drugs from the blood into the retina. This work is adapted from [22], used under CC BY 4.0.

The eye’s unique structure and sensitivity present significant challenges to drug delivery [21]. Over the past decade, numerous studies aimed at advancing the treatment of diverse ophthalmic diseases have highlighted both the challenges of ocular drug delivery and the need to overcome its limitations. Figure 1 illustrates the barriers to ocular drug delivery. Generally, when introducing barriers to ocular drug delivery, they are divided into anterior and posterior sections based on the anatomical structure of the eye. However, this approach does not provide an effective framework for understanding which strategies are most efficient for addressing these challenges. Therefore, we suggest a new classification that departs from the method of identifying ocular drug delivery barriers by the anatomy of the eye. We discuss the major barriers that researchers consider: (1) barriers to intraocular absorption, (2) barriers to movement, and (3) the manifestation of effects at the target site.

### 2.1. Barriers to Drug Absorption into the Eye

Unlike other organs, the eye possesses inherent barriers that restrict the adsorption of medications, such as lacrimation, nasal discharge, and involuntary blinking [21,23]. Research on tear turnover rates (TTR) during basal and reflex blinking has shown that basal TTR ranges between 10–20 (%/min), whereas reflex TTR varies from 31.5–100 (%/min), highlighting the substantial impact of reflex blinking on tear circulation [24,25]. Furthermore, the nasolacrimal duct serves as the entry point for drugs removed through the tear cycle. Snibson et al. conducted an evaluation of the corneal residence time for components present in sodium hyaluronate solution, assessing their behavior and interactions in the context of ocular applications [26]. Although there were slight differences based on the composition of eye drops, the quantity of medication remaining on the ocular surface diminished over time for all tested eye drops. Simultaneously, the drug volume within the nasolacrimal duct and the lacrimal sac was increased, irrespective of the eye drop formulation. The amount of eye drops dissipates rapidly, accounting for approximately 20% of the overall drug present in the lacrimal sac within a minute of administration. This loss of medication to the nasolacrimal tract is considered undesirable due to the potential for systemic absorption, as the lacrimal sac and nasolacrimal duct are both vascularized [1,27]. Direct exposure to the systemic circulation at these sites raises concern about the unintended consequences of drug administration. In research conducted by Müller et al., when medication was administered during the presence of nasolacrimal occlusion (NLO), a 67% reduction in systemic absorption and a 33% extension in drug retention within the anterior chamber were observed [28]. Hence, to overcome the ocular drug absorption limitations posed by the inherent barriers and loss mechanisms associated with the eye, the development of effective ophthalmic drug delivery systems requires multidirectional research and innovations.

### 2.2. Barriers to Movement to the Target Site

Multiple anatomical impediments hinder drug absorption in the eye, as drug particles are required to traverse obstacles to reach the target region after tear circulation [29]. These defensive mechanisms include membranes and blood vessel barriers that protect the human eye from external substances. Although nanoparticles, due to their small size, can typically navigate these barriers, they do not fully resolve all challenges [30]. The primary contributing factors to these obstacles are the mode of penetration and the barrier components.

Cells typically have intercellular spaces or gaps to maintain fluid continuity. The paracellular pathway, wherein drugs penetrate these intercellular spaces, poses challenges for drug absorption and delivery, owing to the strong binding forces. This intercellular binding acts as an anatomical barrier component in the eye, impeding the entry of indiscriminate substances [31]. Barrier mechanisms maintain and regulate cell-to-cell adhesion, which can be broadly categorized into four groups: tight junctions, adherens junctions, gap junctions, and desmosomes. Tight junctions and gap junctions function as molecular gates, whereas adherens junctions and desmosomes provide cell-to-cell adhesion and structural stability without directly influencing material permeation, but affecting cell clearance.

Tight junctions, adhesive sites between adjacent cells, regulate substance movement by sealing gaps and are crucial in epithelial and endothelial cells. These junctions function to protect the body from external factors and prevent external substance intrusion [32]. The cornea, a prime example of intraocular tissue containing tight junctions, has an epithelial barrier that safeguards ocular tissue from infections and restricts molecular movements [33]. Composed of intricate protein networks, tight junctions involve key components such as claudin, occludin, jetril, and zorin, which play crucial roles in establishment and maintenance. These protein interactions create a narrow extracellular matrix space and allow only minimal transport of substances, particularly important in structures such as the blood–retinal barrier (BRB) [34]. BRB tight junctions effectively prevent salts, extracellular materials, and toxic substances from intruding retinal tissues and cells, ensuring normal eyeball function. Shen et al. have demonstrated that exposure to transforming growth factor (TGF-β1) increases paracellular permeability in retinal microvascular endothelial monolayers [35]. TGF-β1 triggers the phosphorylation of tyrosine in VE-cadherin and claudin-5 proteins, constituents of inner BRB tight junctions, highlighting the impact of these junctions on material permeability.

Gap junctions, a type of cell adhesion structure, facilitate direct electrochemical interactions between cells through tiny channels created by hexameric proteins called connexins [36]. These channels enable the transfer of small-scale information and materials, including ions, minute quantities of sugars, neurotransmitters, second messengers, and small amounts of metabolites. However, the 1.5–2-nanometer inner diameter of connexin hexamers poses a challenge for the passage of larger materials such as polymers. Trementozzi et al. experimented with dextran molecules of various molecular weights to assess their ability to traverse gap junction channels [37]. They found that dextran with molecular weights of 20 and 40 kDa passed through all six cell layers, whereas 70 kDa dextran only moved across two cell layers. These results indicate a decreased capacity for dextran to travel through gap junction channels as its molecular weight increases, consistent with expectations. Gap junction networks are not active in all cells like HeLa cells, but they play a significant role in ocular cells and should not be overlooked. In fact, it was found that only 2–3% of the dye was transmitted through gap junctions in HeLa cells, regardless of the presence or absence of carbenoxolone (CBX), a gap junction inhibitor. However, in RPE cells, only 3% of the dye was transferred when CBX was present, compared to 40% of the dye when CBX was absent [38]. These results highlight the presence of active, functional gap junctions between RPE cells and underscore the significance of gap junctions in ocular drug delivery.

### 2.3. Barriers to Effect Manifestation at the Target Site

Indeed, overcoming the barrier posed by the cell membrane is a crucial aspect of drug delivery, not just for ocular applications, but for all types of drug delivery within the body [39]. Nonetheless, in the context of ocular drug delivery, reaching the target site itself presents a significant challenge, making the drug’s efficacy at the target location a crucial factor. Consequently, understanding the barriers impeding drug passage becomes vital for optimal delivery outcomes. The cell membrane controls the movement of substances into and out of cells, and failure to overcome this barrier can significantly reduce the bioavailability and effectiveness of the drug. Several factors influence the permeation of drug molecules across the cell membrane, including the material properties of drug molecules, cell membrane structure, properties, and the presence of transporters [40]. Developing an effective ocular drug delivery system requires careful consideration of these factors to ensure that drug molecules can successfully permeate the cell membrane and exhibit their intended therapeutic effect.

#### 2.3.1. Lipid Bilayer of Cell Membranes

Cell membranes, primarily composed of lipids, present difficulties for substances with varying chemical properties, particularly hydrophilic or lipophilic, to pass through. Some lipophilic substances can enter cells via the intracellular lipophilic pathway, which involves separating and transporting polar lipid molecules present on the cell membrane’s periphery [41,42,43]. The maximum size of lipid-soluble molecules that can pass between cells via the lipophilic route is around 400 Da [44,45], as larger molecules struggle to interact with the lipid bilayer and encounter interferences. Researchers have attempted to transform water-soluble substances that are unable to cross cell membranes into fat-soluble ones [46,47]. However, alterations in lipid solubility often negatively affected permeability by either reducing a molecule’s affinity for its target receptor or increasing its size beyond 400 Da.

Retinal pigment epithelial (RPE) cells exhibit higher lipid content compared to other cells due to their close interaction with photoreceptors, which relies on lipid-dependent processes. RPE cells have a lipid content of 55–60%, higher than the 40% observed in red blood cells [48,49]. This elevated lipid content renders RPE cells relatively resistant to the penetration of hydrophilic substances. However, it can also be hypothesized that this property facilitates the cellular uptake of chemicals via lipophilic pathways. Studies indicate lipophilic molecules have an advantage over hydrophilic molecules when traversing the RPE, but this advantage applies only to low-molecular-weight lipophilic molecules [50,51]. The composition and unique properties of RPE cells contribute to their distinct behavior, particularly in drug delivery and pharmacology.

#### 2.3.2. Receptor for Cell Membrane Penetration

In addition to the lipophilic route, drug molecules often necessitate a ligand to cross cell membranes. Receptor proteins, commonly found in cell membranes, exhibit strong affinities for specific chemicals (or ligands). Ligand binding alters the receptor structure, enabling the complex to efficiently pass through the cell membrane. However, some basic drugs might struggle with this process due to the insufficiency of the appropriate chemical structures or functional groups needed to act effectively as ligands. The binding of drug molecules and receptors is not dictated by a single mechanism, but is elicited through a multitude of interactions involving varying chemical structures and functional groups [52]. Additionally, even with the same mode of binding, the degree of interaction can diverge, influenced by diverse molecular characteristics such as shape, size, and polarity [53,54]. Therefore, the incorporation of ligands, with strong interactions with receptors, could potentiate the engagement between the drug and the receptor compared to its intrinsic interaction. Regardless of ligands, transcytosis may be induced by certain receptors without a ligand, enabling cell membrane penetration via the potential difference created by ion charges. Nonetheless, this process often requires energy expenditure, and without sustained chemical energy, adsorptive transcytosis is unfeasible. Balancing energy expenditure and drug efficacy presents a challenge for efficient drug delivery.

Receptors on intraocular cells may offer valuable insights for drug delivery strategies. For instance, P2Y receptors on retinoblastoma cells are metabolic G-protein-coupled receptors involved in cell function. The activation of these receptors can stimulate RPE fluid pump function, leading to a temporary enhancement in fluid uptake [55,56]. This increased uptake may also prove effective for the incorporation of drug nanoparticles. By utilizing receptor-mediated cellular entry, it is possible to augment the influx of drugs into cells. Consequently, it is essential for drug molecules to possess a suitable ligand that can facilitate this process. Harnessing receptor–ligand interactions offers a promising approach to optimize drug delivery and improve the efficacy of treatments for ocular disorders.

## 3. Recent Progress and Challenges in Overcoming Biological Barriers with Nanomaterials

In recent years, nanotechnology has provided new ideas and strategies for treating ophthalmic diseases by improving penetration, achieving controlled release, and improving bioavailability, as well as reducing irritation and even achieving targeting in the field for drug delivery research. With the benefit of size, nanoparticles can potentially penetrate the cornea and reach the deeper layers of the eye, where they can deliver drugs directly to the affected tissues with increased permeability [57,58]. Similarly, nanoparticles can also potentially cross the blood–retinal barrier, which is a specialized barrier that protects the retina from harmful substances in the bloodstream.

However, it is essential to note that not all nanoparticles are able to cross these barriers, and even those that can may face challenges such as clearance by the immune system and potential toxicity [59]. Therefore, the development and use of nanomedicine for ocular drug delivery require careful consideration and evaluation to ensure safety and efficacy.

The distinctive properties of carriers are given top importance in the formulation of nanopharmaceuticals. The effectiveness of medication delivery is significantly influenced by the choice of an appropriate carrier from among the numerous varieties, including those mentioned in Table 1. For instance, hyaluronic acid (HA), a stimuli-responsive gel material, is widely utilized for its capacity to form efficient drug delivery systems [60,61]. These hydrogels possess the ability to encapsulate therapeutic agents through volumetric shrinkage and increased solubility while maintaining an aqueous environment and providing a controlled gelation time with extrinsic sheath effects. Due to these properties, they can effectively deliver poorly water-soluble drugs in a stable manner.

Recent advancements in nanocarriers have shifted the focus towards enhancing their activity, moving beyond solely considering the carriers’ inherent characteristics [62]. This is achieved by modifying their physicochemical properties, such as binding different materials to nanocarrier surfaces. Overall, optimizing the physicochemical properties and surface modifications of nanocarriers can significantly contribute to overcoming ocular drug delivery barriers [63]. The choice of modification method is often connected to the targeted drug delivery barriers, as there are multiple challenges that must be overcome for successful drug delivery. These advancements enable a further efficient, targeted, and safe transport of therapeutics within the eye, which can ultimately lead to improved outcomes for patients with several ocular conditions.

**Table 1:** Pros and cons of prior developed ocular drug delivery systems.

Delivery Systems	Pros	Cons	Ref
Liposomes	Sustained drug release, improved bioavailability, biodegradable, biocompatible, and non-immunogenic	Poor stability, leakage, and fusion of drugs	[64,65]
Solid lipid nanoparticles (SLNs)	Drug loading capability for lipophilic and hydrophilic drugs, suitable for autoclaving sterilization, increased ocular bioavailability, and prolonged ocular retention time	Drug expulsion following polymeric transition during long storage	[66,67]
Polymeric nanoparticles	Increased ocular penetration, prolonged residence time, and simplicity of change	Burst effect and aggregation of particles and toxicity	[68,69,70,71,72]
Dendrimer	Improved drug penetration and effectiveness	Blurred vision and loss of eyesight	[73,74,75,76]
Stimuli-responsive gel	Sustained drug release, improved biocompatibility, and biodegradability, Versatility in design	Limited to smaller molecular weight drugs, poor stability, and temperature sensitivity (difficulty in retaining water)	[77,78,79]
Inorganic nanoparticle	Improved ocular penetration by small size, controlled release by physical and chemical properties (super-magnetism, photothermal, etc.)	Poor stability, bioavailability	[80,81,82,83]

### 3.1. Continual Strategies in Indirect Modulation

In the early stages of surface modification research for nanomaterials, the focus was on increasing or decreasing interactions with nanomaterials based on the properties of materials within the body [84]. Modifying nanomaterials using their functional groups, ionicity, or other inherent characteristics was considered relatively simple compared to employing specific targeting molecules, which require multiple steps such as complex synthesis, purification, and coating. Furthermore, since such modifications can potentially be applied to body tissues with similar properties, their application range appeared broad. These types of studies that are aimed at improving the properties of ocular drug-releasing carriers continue to be conducted even today. However, despite the advantages mentioned above, these modifications are not currently considered as effective as those that involve using targeting molecules. This is primarily because the focus of drug-releasing carrier technology has shifted towards targeting potential, which offers a higher degree of precision and selectivity in delivering therapeutics to desired tissues or cells. Though the targeting molecules strategies increase value in ocular drug delivery, non-targeted modifications still hold value in certain contexts for an advanced, efficacious, and safe approach to drug delivery [85]. By tailoring nanocarriers to selectively interact with particular targets in the body, it is possible to achieve greater drug release efficiency, minimize off-target side effects, and optimize treatment outcomes for patients with various ocular conditions and other medical conditions broadly.

#### 3.1.1. Leveraging Mucoadhesion for Effective Drug Adsorption

**Figure 2:**
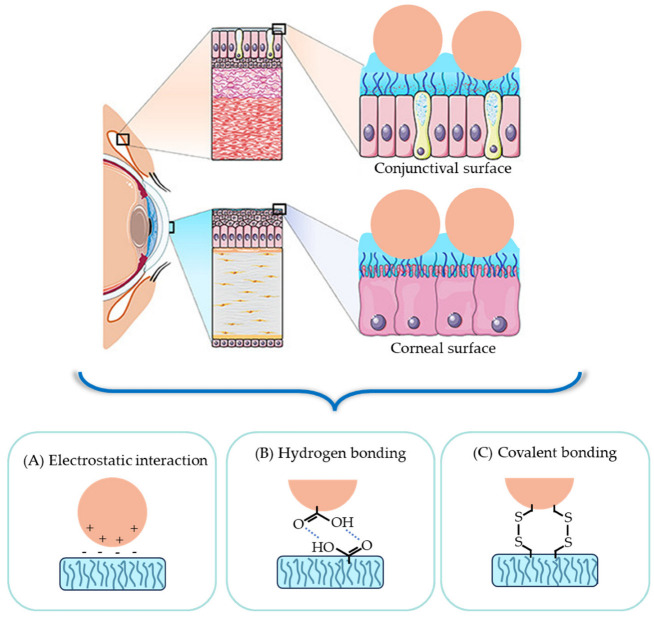
Scheme for mechanisms of interaction between mucins and nanocarriers on the corneal and conjunctival surface; (**A**) electrostatic interaction, (**B**) hydrogen bonding, (**C**) covalent bonding. This work is adapted from [87], used under CC BY 4.0.

Mucoadhesion indicates adhesion to the mucosal surface, improving precorneal residence time. The tear turnover mechanism, which acts as a defense system against external chemical or biological stimuli, is one of the most significant factors that contributes to the loss of drug bioavailability. Nanoformulations help to overcome problems related to the solubility and permeability of the drug, but are unable to withstand mucociliary clearance. Therefore, utilizing mucoadhesive polymers that adhere to the mucin layer coating the corneal surface of the eye through attractive interactions, such as electrostatic interaction, hydrogen bonding, and covalent bonding, could be a promising strategy [86]. This principle is illustrated in Figure 2. Mucoadhesive polymers can also protect nanoparticles from clearance by tears or the immune system, further enhancing their therapeutic efficacy.

- Surface modification of electrostatic interaction with mucin

**Figure 3:**
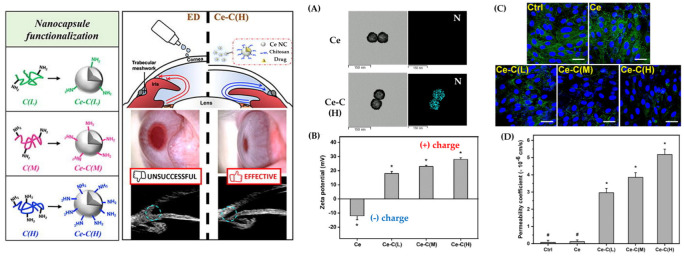
Chitosan coating, which results in cationic properties, effectively enhances cell permeation. (**A**) TEM images and EDS analyses, with or without chitosan coating, confirm that the nitrogen component is detected when chitosan is coated. (**B**) Upon chitosan coating, the zeta potential value transitions from negative to positive. (**C**) Confocal laser scanning microscopy images of immunofluorescence-stained cell layers show cerium (Ce) nanocrystals and chitosan-coated Ce nanocrystal samples (scale bar: 50 μm). (**D**) Transepithelial electrical resistance (TEER) values of nanocarriers across the cell layer (* *p* < 0.05 vs. *all groups*; # *p* < 0.05 vs. *NC and PC groups*; n = 4) indicate that chitosan coating leads to significant cell permeation enhancement. Reprinted from [89], © 2023 Elsevier Ltd. (Amsterdam, The Netherlands). All rights reserved.

Mucin, a major component of mucus on the mucosal surface, can easily electrostatically interact with substances that exhibit cationic properties due to a negative surface charge. Numerous cationic polymers have the potential to function as adhesive polymers through electrostatic attraction. Chitosan, a cationic hydrogel polymer, is widely used among these polymers. This is due to the fact that chitosan has great biocompatibility and includes an amino group that may be readily protonated [88]. Recently, Nguyen et al. prepared pilocarpine-loaded ceria nanogel (Ce NC) coated with chitosan hydrogels for ocular application [89]. The Ce NC was first PEGylated to facilitate the formation of an amide bond, and subsequently coated with chitosan hydrogel via the amide bond. The study indicated that the functionalization of nanocarriers with chitosan hydrogel does not affect their physical capacity for drug storage. Moreover, it was found that the zeta potential of Ce NC can be modulated by chitosan hydrogel coating, and the high amination level of chitosan can have a higher number of amino groups, resulting in an increased magnitude of positive surface charge (Figure 3B). This can also be confirmed through transmission electron microscopy (TEM) and EDS mapping images (Figure 3A). In addition, the penetration ability of Ce NCs encoded with chitosan hydrogel was improved by about 43 times in the case of chitosan, which had the highest amination level (Figure 3D). The free amine group of chitosan’s backbone has a significant effect on the swelling behavior of chitosan hydrogel, so depending on the level of amination, it can also be utilized as a pH-responsive release. In another study, polycationic chitosan oligosaccharide (COS) was synthesized in an attempt to improve the efficiency of ocular drug delivery through surface linkage with nanostructured lipid carriers (NLCs) [90]. NLC particles coated with COS hydrogel showed a 2.4-fold increase in penetration compared to uncoated NLC. In addition, 86.9% of the COS-based formulation was still present on the corneal surface for up to 10 min, which is 2.02 times higher than non-coating. Furthermore, it was demonstrated that the in vivo efficacy substantially increased under neutral or mildly alkaline pH conditions similar to those found in tear film. From the results obtained, it was demonstrated that cationic hydrogel polymers such as chitosan and its oligosaccharides can provide excellent mucoadhesive properties due to ionic interactions with the charged sialic acid residues (negatively charged amino groups) of mucins.

- Harnessing hydrogen bonds with carboxyl groups in mucin for nanocarrier surface modification

Polymers including functional groups that can generate hydrogen bonds with carboxyl groups of mucin glycoproteins are being used to improve mucoadhesive properties in addition to electrostatic attraction exploiting the anionic nature of mucins. Hydrogels composed of hyaluronic acid, a linear polysaccharide molecule characterized by the presence of carboxyl and hydroxyl groups, serve as an example of such polymers. Hydrogen bonding between the hydroxyl group of hyaluronic acid and the carboxyl group of mucin glycoproteins strengthens the mucoadhesive properties of hyaluronic acid. Landucci et al. showed that hyaluronic acid-coated liposomes could be effective in the treatment of dry eye syndrome [91]. Regarding mucoadhesive properties, evaluated based on zeta potential, the interaction between hyaluronic acid-coated liposomes and mucin increased, strengthening the negative zeta potential of mucin due to the negative charge of hyaluronic acid. In another study, chemically modified thiolated hyaluronic acid hydrogels with a sulfhydryl-containing ligand showed improved biocompatibility and retinal adhesion [92,93]. Additionally, hyaluronic acid hydrogels have binding properties with the CD44 cell surface receptor, which can lead to improved adhesion in ophthalmic cells, especially in human conjunctiva and corneal epithelium and retinal pigment epithelium. These factors make hyaluronic acid hydrogels especially attractive as polymers that can improve therapeutic effectiveness. Other hydrogels that can form hydrogen bonds with mucin glycoproteins to increase mucoadhesiveness include pullulan and alginate hydrogels.

**Figure 4:**
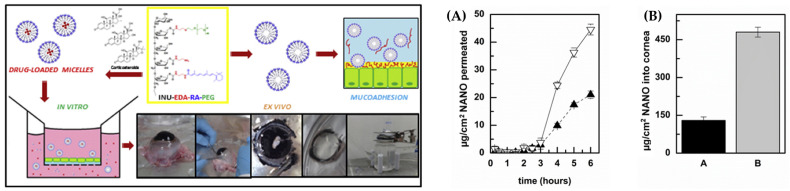
The adhesion and permeation of mucin were enhanced through PEGylation. Micelles loaded with drugs are utilized for in vitro experiments, while the same micelles without drug loading were used for ex vivo mucoadhesion-related tests. Figure (**A**) shows the ex vivo transcorneal permeation profiles of self-assembling nanoparticles (▽, solid line) and INU-EDA-RA (▲, dash line) systems in terms of the μg/cm^2^ of permeated nanoparticles (±SD) as a function of incubation time (h). In Figure (**B**), the μg/cm^2^ of INU-EDA-RA (column A) and self-assembling nanoparticles (column B) into corneal tissue at the end of the experiment are presented. The PEG-coated self-assembling nanoparticles exhibited over 2-fold more excellent transmittance than the non-PEG-coated particles. Reprinted from [95], © 2019 Elsevier Ltd. All rights reserved.

In addition to the aforementioned hydrogel polymers, there are other polymers that impart mucoadhesive and transdermal penetration-enhancing properties based on their biocompatible characteristics. Polyethylene glycol (PEG) and polyvinyl alcohol (PVA) serve as notable examples [94,95]. Di et al. demonstrated that the PEGylated nanosystem showed an approximately 2-fold increase in permeability compared to other nanoparticles, as depicted in Figure 4. Nevertheless, in the case of synthetic polymers, the influence on transdermal penetration was generally noticeable, whereas the effects on mucosal adhesion were inconsistent, with certain instances exhibiting no significant results [96,97]. This phenomenon may be attributed to weaker bonding forces compared to other types of bonding interactions. The improvement of mucosal adhesion via hydrogen bonding relies on the dipole–dipole interactions between partially positively charged hydrogen atoms and partially negatively charged atoms, such as oxygen or nitrogen. In fact, compared to that based on electrostatic interactions, the beneficial effect of PEGylation on mucosal adherence is up to five times smaller [98]. To compensate for this, other bonding methods such as covalent bonding are being attempted. Using PEGylated liposomes adorned with maleimide, Roman et al. showed better retention on the conjunctiva in drug administration [87]. Because mucins include thiol groups, maleimide can display greater performance by forming a covalent link with these groups. Additionally, research has demonstrated that thiolated PEG coatings considerably enhance mucoadhesiveness when compared to conventional PEG coatings [96].

- Utilizing covalent bonds with cysteine-rich subdomains in mucin for nanocarrier surface modification

**Figure 5:**
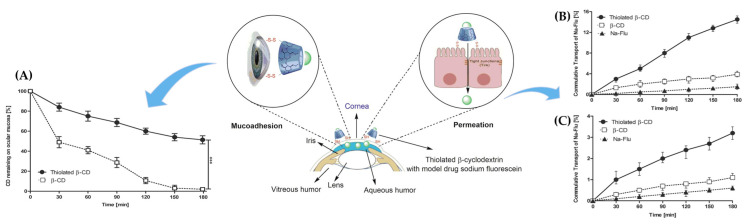
Disulfide bond formation with mucin by thiol groups and permeability enhancement. (**A**) Retention time comparison between thiolated and unmodified nanoparticles in porcine ocular mucosa. Values are presented as the mean ± standard deviation of triplicate experiments (*** *p* < 0.001). Drug transport through the porcine ocular mucosa, including (**B**) the conjunctiva and (**C**) the cornea. Transport data are expressed as the percentage of the total drug dose using 0.5% (*m*/*v*) unmodified nanoparticles and 0.5% (*m*/*v*) thiolated nanoparticles application. Reprinted from [100], used under CC BY 4.0.

As mentioned above, polymers with thiol groups as their backbone, called thiolated polymers or thiomers, are capable of interacting with mucin cysteine, which has sulfhydryl groups, forming disulfide bonds [99]. This method of interaction is particularly noteworthy due to the much stronger adhesion it provides compared to the ionic and hydrogen bonds. Asim et al. synthesized thiolated β-cyclodextrin (CD) by replacing all primary hydroxyl groups (-OH) of the CD backbone with thiol groups (SH), and investigated its mucoadhesiveness and permeability [100]. They found that the mucosal adhesion of the thiolated β-CD to ocular mucosa increased by 26 times compared to unmodified CD, with over half of the thiolated β-CD remaining attached to the mucosa after a 3-h test. This was attributed to the formation of a disulfide bond between the cysteine-rich mucous glycoprotein and thiolated β-CD. Furthermore, the effects of thiolated β-CD on sodium fluorescein permeation in the conjunctiva, sclera, and cornea were investigated and found to increase, suggesting an increased corneal residence time and opening of the tight junctions by thiolated β-CD. These results are represented in Figure 5. Similar results were obtained in other studies [101,102,103].

However, this method is not applicable to drugs with sulfhydryl groups and/or disulfide bonds, including captopril, tiopronin, omapatrilat, and desmopressin. Moreover, unprotected thiomers lose their reactivity towards mucous glycoproteins and their viscosity may increase in situ, preventing the polymer from penetrating the mucous gel layer, particularly at pH values above 5 [104,105]. To address these issues, recent studies have investigated a new type of covalent mucin-binding polymer that can form an amide bond with the amino groups, including lysine and arginine substructures of mucin glycoproteins, in addition to the thiol group of the cysteine-rich subdomain. Menzel et al. developed a polymer backbone of polyacrylic acid coupled with N-Hydroxysuccinimide (NHS) and confirmed its mucoadhesive properties based on covalent bonds with amino groups [106]. The high-molecular NHS ester reacted selectively with mucosal amino groups and showed sufficient stability in the buffer solution (pH 6.8). Unlike thiomer-based mucosal adhesion, high-molecular NHS esters can be used on a variety of surfaces, such as lysine-rich connective tissue and muscle tissue, and are also effective for drugs with sulfhydryl groups. This area of research is expected to continue to develop and find numerous applications in the future.

#### 3.1.2. Optimizing Cellular Penetration and Uptake through Surface Interaction Adjustments

In the past, the understanding of specific complexes or materials constituting barriers was comparatively limited in contrast to our current knowledge [107]. Moreover, targeting certain molecules was approached with caution, as the loss of function could lead to severe side effects, such as carcinogenesis. As a result, researchers focused on the development of cell-to-cell penetration and cell-opening technologies that were based on relatively non-specific modes of action. Such a strategy involves inducing the controlled and reversible opening of specific proteins present in cell membranes and cell junctions, which are also considered physiological barriers. By manipulating the interactions of these proteins, it is possible to increase the safe and effective movement of nanocarriers and improve intracellular drug delivery. This approach continues to be utilized in recent research and holds promise for advancing drug delivery systems, particularly as our understanding of cellular barriers and targeting mechanisms further improves.

- Transforming junction protein–nanocarrier interactions for improved paracellular permeability

The 1993 discovery of occludin, the first tight junction integral membrane protein, sparked the creation of methods for increasing paracellular permeability [107]. It can be difficult to improve paracellular route barriers such as tight junctions and gap junctions utilizing a nonspecific method of action. Since this pathway’s principal function is passing the gap bridging the intercellular, expanding it is the only practical approach to increase its permeability. Typically, modulators directly affecting proteins forming tight junctions or gap junctions are employed to expand the intercellular space [108]. However, similar effects can also be achieved through simple surface modifications of nanocarriers. Cationic polymers are one method for enhancing the paracellular route by nanocarrier surface modification.

In models of epithelial cells, cationic polymers such poly-l-lysines, polyethyleneimine, and chitosan may cause the reversible opening of tight junctions. This happens when one of the primary proteins that make up tight junctions, the high molecular weight zonula occludens toxin (Zot), interacts with cationic polymers to momentarily open tight junctions. By interacting with molecules including claudin, occludin, and JAM-1 (Junctional Adhesion Molecule), the Zot protein triggers the release of tight junctions. Chitosan and chitosan-N-acetylcysteine (CS-NAC) have been studied for their effects on conjunctival epithelial cells by Schuerer et al. Chitosan entered conjunctival epithelial cells in vitro [109]. Within 12 min of instillation, the polymer had permeated the tissue and was visible not only in the conjunctival epithelial cells on the surface, but also in cells that were 80 μm below the surface. Additionally, transepithelial electrical resistance assay findings showed how chitosan affected the conjunctival epithelial cells’ ability to operate as a barrier, showing a 60% decrease in electrical resistance values after up to 60 min of incubation. However, CS-NAC was not detected within the conjunctival epithelial cells, and instead formed a 3D network on the cell surface. This outcome is anticipated, as the size of CS-NAC imposes constraints. Furthermore, a 24-h observation of the opened tight junctions in conjunctival epithelial cells by chitosan revealed that remodeling was challenging. The prolonged opening of tight junctions may disrupt cellular equilibrium and stability, making it crucial to examine this aspect. Additionally, even when coated with a substance such as chitosan, if size limitations persist, the desired effect may not be achieved, warranting careful consideration of this factor.

- Transforming cell–nanocarrier interactions for improved transcellular permeability

Indeed, cell membranes are selectively permeable to substances that have an affinity for specific ligands and are recognized by them. Ligand–receptor interaction is not always required for substances to cross the cell membrane, as appropriate passage can be achieved by modulating the interaction with the cell membrane itself [110]. This can involve altering the physicochemical properties of the substance, such as lipophilicity, charge, and size, to improve or control its permeability. Different strategies, including the use of nanoparticles or the modification of drug carriers, can also be employed to improve the interaction with the cell membrane and facilitate the passage of substances through the membrane.

PEGylation is a critical strategy for regulating the efflux and permeation capabilities of representative nanocarriers. By attaching PEG (polyethylene glycol) polymers to the surface of nanoparticles, it improves uptake into cells by blocking the action of hydroxyl groups present on the surface of nanoparticles. Generally, cells exhibit relatively high selectivity for substances that pass through the cell membrane and interact with the extracellular matrix or cell surface proteins; these substances have the potential to be actively processed through negative pathways, such as cell erosion. If nanoparticles remain outside the cell without contacting the cell membrane due to cell corrosive effects, they may not be absorbed into the cell and may not provide effective drug delivery. PEGylation reduces cell corrosive effects by preventing nanoparticles from binding to cell membranes, thereby facilitating uptake into cells. Mun et al. investigated the corneal barrier properties using PEGylated nanoparticles [111]. Their study of the penetration of PEGylated nanoparticles into de-epithelialized ocular tissues revealed that the interaction between the corneal surface and the nanoparticle’s thiol groups plays a substantial role in penetration, as compared to the effect of particle size. Additionally, small molecule PEGylated nanoparticles remain on the ocular surface due to their adhesive properties, and PEGylation using high molecular weight PEG masks most of the thiol groups on the nanoparticle surface, allowing PEGylated nanoparticles to pass through the substrate. This confirms that PEGylation is an effective strategy to decrease the interaction between nanoparticles and cells and improve cell permeation function.

The zwitterionic coating is another way to weaken the interaction between nanoparticles and cell membrane surfaces. By creating a structure in which carboxyl and ammonium functional groups are combined on the nanoparticle surface, subtle charges are generated, as if positive and negative charges coexist simultaneously. This neutral property enables nanoparticles to minimize interactions with cell membranes. Additionally, the interaction force between particles increases, and the degree of particle dispersion rises, making passage through cell membranes efficient. In particular, zwitterionic coating possesses a biomimetic motif (positive ion unit), providing biocompatibility and a “stealth” effect similar to PEGylation, which helps nanoparticles be less likely to be recognized as foreign substances [112]. Based on this concept, Ma et al. constructed zwitterionic micelles and studied their transport and drug release at tumor sites [113]. Zwitterionic micelles were able to reduce breakage due to non-specific protein adsorption, leading to high anticancer efficacy in tumor tissue. Compared to the free drug, drugs loaded into zwitterionic micelles demonstrated approximately 30% improved tumor inhibition. The modification effect with zwitterionic substances has been proven, and ocular drug delivery studies are currently being explored. However, further research is necessary to determine whether zwitterionic modification has a positive impact on intraocular cell membrane penetration.

### 3.2. Improving Nanocarrier Drug Delivery through Specific Ligands as Modulators

Investigations into passive surface modifications that regulate interactions with biological systems based on material properties have demonstrated promising outcomes. However, recent advances in drug delivery technology have prompted a shift towards the use of specific modulators, including proteins, peptides, and aptamers [114,115,116]. These targeting molecules confer substantial advantages over non-targeted modifications by enhancing precision, selectivity, and potency in therapeutic delivery. Certain modulators facilitate tailored interactions with desired targets in the body, potentially leading to efficient drug release at the intended site and optimized treatment outcomes. As drug-releasing carrier technology continues to progress towards exploiting targeting potential, the employment of targeting molecules will increasingly contribute to the development of active drug delivery systems that bolster both therapeutic efficacy and safety.

#### 3.2.1. Proteins and Short Peptides

Ligand–receptor reactions and antigen–antibody reactions are familiar molecular interaction reactions that can boost delivery into cells. Both antibodies and ligands are proteins produced in the body, making them target molecules with high biocompatibility. Though all target molecules can be used for nanocarrier surface modification, the nanocarrier for ocular tissues is often attempted with smaller protein molecules, considering that antibody size can make it difficult to pass through cells [117]. Transferrin is an iron transfer protein in the body, and studies have shown that transferrin receptors on retinal pigment epithelial cells are upregulated in diseases of the eye’s posterior segment, such as vitreoretinopathy, glaucomatous neuropathy, and age-related macular degeneration. As a result, retinal-targeted drug delivery using the reaction between the transferrin receptor and transferrin present on the cell surface is being studied [118,119,120,121]. When transferrin binds to its receptor on the cell surface, the complex containing transferrin is internalized and can easily enter the cell. This was demonstrated in a study by Singh et al., which compared the retinal delivery and drug effects of PLGA nanoparticles (NPs) functionalized with transferrin to those without [122]. The functionalized NPs showed both enhanced retinal delivery and increased intra-receptor drug expression within retinal vascular endothelial cells, photoreceptor outer segments, and RPE cells. Another study found that nanocarrier size and the presence or absence of transferrin could alter ocular barrier permeability [117]. Despite being with transferrin, large-sized liposomes could not pass through the membrane pores and remained on the choroidal side. In contrast, small-sized transferrin-liposomes were able to be delivered to the RPE, whereas small liposomes without transferrin failed to show fluorescence signals in RPE. These findings support the idea that active targeting with protein molecules can be highly effective for efficient delivery to the target tissue. However, they also highlight the importance of drug delivery particle size.

**Figure 6:**
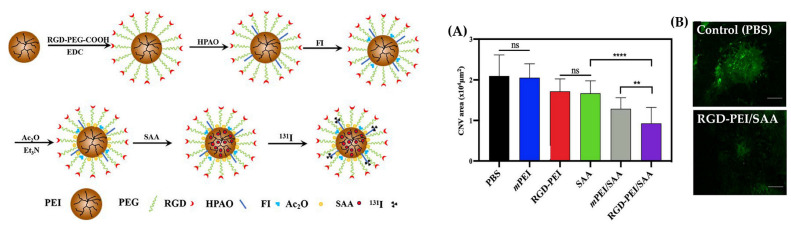
Disulfide bond formation of RGD peptides with mucin by thiol groups and permeability enhancement. (**A**) Quantitative analysis of the average CNV area (mm^2^) in each group (n = 28). RGD-modificated nanoparticles reduced neovascularization in CNV mice model. The error bar stands for the standard error of the mean (** for *p* < 0.01, **** for *p* < 0.0001, ns: no significance). (**B**) Representative images of RPE/choroid/sclera flat mounts with isolectin B4 staining from laser-induced CNV, following the intravitreal injection of each type of nanoparticle. Reprinted from [124], used under CC BY 4.0.

In addition to small proteins such as transferrin, short forms of peptides are indeed utilized for targeted drug delivery. Peptides, such as ATWLPPR, target specific receptors such as VEGFR-2. Li et al. conjugated ATWLPPR to nanoliposomes loaded with pigment epithelial-derived factor (PEDF), an angiogenesis inhibitor [123]. Due to the interaction between ATWLPPR ligand and the VEGFR-2 receptor, PEDF was able to bind exclusively to choroidal neovascularization and efficiently move into the cell’s cytoplasm. Furthermore, peptides such as RGD (arginine-glycine-aspartate) are being explored to improve cell membrane passage based on their binding with extracellular matrix proteins. Proteins such as fibronectin, vitronectin, and laminin in the extracellular matrix contain RGD sequences, which can increase their interactions with integrin receptors in cells. Zhang et al. demonstrated that targeted antiangiogenic therapy could be performed based on the binding of RGD-modified multifunctional nanoparticles to integrin α_v_β_3_, which is overexpressed in the CNV membrane of the RPE layer [124]. Some results related to these findings are depicted in Figure 6. Utilizing peptides other than proteins such as transferrin, which can bind to receptors present in cell membranes, can maintain the advantages of targeting without increasing the size of nanocarriers due to surface modification. This approach provides a valuable strategy for enhancing effective and selective drug delivery within the cellular environment.

Though the application of antibodies for surface modification of nanocarriers in ocular drug delivery has been relatively unexplored, antibodies have been utilized for this purpose in other ways. One notable example is antibody-drug conjugates (ADCs), which involve directly binding an antibody to a drug, serving as a drug carrier. The initial ADC designs targeted cancer treatment and showcase their potential to reduce toxicity compared to conventional small molecule cancer therapies [125]. With the expanding scope of ADC research for ocular disease treatment, concerns regarding non-target drug toxicity were addressed, and drug delivery to hard-to-reach areas in the posterior segment, such as the choroid, became possible. This can be attributed to the small form of ADCs, which augments cell-to-cell permeability, and the antibody reaction, which improves entry into cells. In a study by Lee et al., an ADC targeting PDGFRβ, a receptor for the angiogenesis-inducing growth factor platelet-derived growth factor (PDGF)-BB, was investigated [126]. Immunohistochemical experiments revealed differences in PDGFRβ expression levels between normal and neovascular vessels, confirming the low toxicity of ADCs targeting mPDGFRβ in both anatomical and functional aspects. The retinal layer thickness ratio remained unchanged, and no significant amplitude differences were observed between the ADC and control groups in the ERG and OptoMotry tests. However, some studies have reported ocular toxicity results associated with ADCs [127,128,129,130]. Although the exact mechanisms underlying the ocular toxicity of ADCs remain unclear, increased adverse reactions have been observed, particularly with maytansinoid- and MMAF-containing ADCs [130]. These findings warrant consideration in future research to develop safer and more effective therapeutic strategies involving ADCs.

#### 3.2.2. Cell-Penetrating Peptides

Cell-penetrating peptides (CPPs) are a class of short peptides that facilitate drug transport across cell membranes and serve as a prominent method for bypassing biological barriers. CPPs are known to penetrate cell membranes either through endocytosis or by disrupting the lipid bilayer of cell membranes [131]. One of the earliest examples of CPPs developed for ocular administration was the ocular delivery peptide (POD) (GGG[ARKKAAKA]4; 3.5 kDa), studied by Johnson et al. POD was used as a carrier to deliver recombinant GFP (green fluorescent protein) to retinal tissue in vivo [132]. This strategy demonstrated that direct conjugation of a CPP with a therapeutic agent, similar to antibody-drug conjugates (ADCs), can significantly improve the solubility, stability, and cell membrane permeability of the drug. The success of CPPs in ocular drug delivery illustrates their potential to overcome the challenges of delivering drugs to targeted ocular tissues, opening up new possibilities for effective and safe ocular therapeutics.

**Figure 7:**
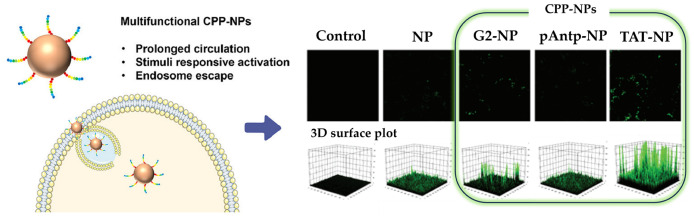
Enhancement of cell membrane penetration and drug efficacy through CPP-NPs. The cellular uptake of nanoparticles (NPs) in the HCE-2 cell line was visualized using green fluorescence. Apart from pAntp-NPs, CPP-NPs exhibited strong fluorescence signals. For pAntp-NPs, fluorescence signals were not detected throughout the eye, but localized to the posterior segment. TAT-NPs and G2-NPs are considered well-suited CPP-NPs for application. This work is adapted from [138], used under CC BY 4.0 and [140] © 2019 Future Medicine Ltd. (London, UK). All rights reserved.

In recent years, studies on modifying nanoparticles (NPs) with cell-penetrating peptides (CPPs) have emerged, demonstrating promising results in diverse diseases such as inflammation, diabetes, cancer, and neurodegenerative disorders [132,133,134,135,136]. The combination of CPPs and nano drug delivery systems has shown potential in overcoming the challenges of drug targeting and delivery, even to the brain, where the presence of the blood–brain barrier excludes nearly all small molecule drugs [137,138]. This suggests that the CPP-NP system could be effective not only in passing through the cell membrane barrier of the anterior segment, but also in the presence of posterior segment barriers such as the blood–retinal barrier (BRB) [132]. In a study conducted by Amit et al., the bioavailability and delivery efficiency of cornea-specific cell-penetrating peptides were enhanced using a gelatin hydrogel-based delivery system [139]. When two peptides, VRF005 and VRF007, were cultured with corneal epithelial tissue lysates, they remained stable for up to 2 h. Both VRF007 and VRF005 exhibited antimicrobial activity at a minimum inhibitory concentration (MIC) of 1 μg/mL, whereas the conventional antimicrobial agent, natamycin, was only effective at 64 μg/mL. Additionally, VRF005 demonstrated antimicrobial activity for up to 4 h, and VRF007 maintained antimicrobial activity for up to 24 h, proving that the peptides improved the performance of the gelatin hydrogel-based delivery system. Another study by Gonzalez-Pizarro et al. modified PEG-PLGA NPs with different CPPs (TAT, penetratin, and antimicrobial peptide G2) and encapsulated fluorometholone (FMT) [140]. Compared to the control group, which showed virtually no signal, the fluorescence signal improved when nanocarriers were utilized. However, whereas penetratin-NPs and unmodified PEG-PLGA-NPs exhibited signals only in the posterior segment of the eye, TAT-NPs and G2-NPs displayed strong signals in both the anterior and posterior segments (Figure 7). At first glance, penetratin-NPs and unmodified PEG-PLGA-NPs may appear as effective carriers for posterior segment delivery. However, when considering anti-inflammatory activity results, the opposite is revealed. Assessments of IL-1β, IL-6, IL-8, and TNF-α cytokines in HCE-2 cells exhibited elevated levels for almost all cytokines in both penetratin-NPs and unmodified PEG-PLGA NPs. In other words, considering that the drug transport outcomes of penetratin-NPs and unmodified PEG-PLGA-NPs stem from compromised tight junctions caused by decreased cell stability, they are rendered unsuitable as efficient drug delivery vehicles.

Previous studies have demonstrated that conjugating cell-penetrating peptides (CPPs) with drugs or drug-loaded nanoparticles can make better cellular membrane permeation and therapeutic efficacy. However, it is important to note that not all CPPs exhibit such effects uniformly, and in certain cases, they may lead to undesirable outcomes. Given these findings, there is a need for comprehensive in vivo investigations to assess the safety and effectiveness of CPPs in drug delivery systems. Such studies will enable researchers to identify potential side effects, including cytotoxicity, immune responses, and off-target activity. Furthermore, these investigations should explore the optimal design of CPP-drug conjugates to maximize therapeutic benefits while minimizing adverse effects. Overall, gaining deeper insights into the various characteristics of CPPs will contribute to the advancement of safer and optimal drug delivery approaches in medical applications.

#### 3.2.3. Aptamers

**Figure 8:**
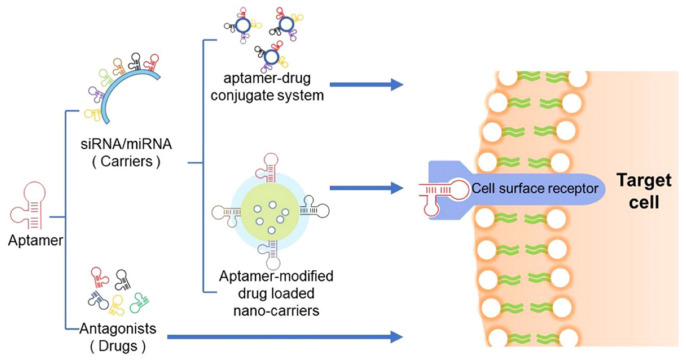
Schematic representation of aptamer application modes in drug delivery research. Reprinted from [63], © 2022 IOP Publishing Ltd. (Bristol, UK). All rights reserved.

Aptamers, short strands of synthetic RNA or single-stranded DNA oligonucleotides, typically consist of several tens of nucleotides and serve as representative active targeting molecules [141]. They are capable of binding target molecules with exceptional specificity and high affinity, akin to antigen–antibody interactions, leading to their nickname as “chemical antibodies” [142]. Ongoing research on aptamer-based drug delivery is expanding due to the myriad of advantages offered by these molecules. The secondary and tertiary folding of aptamers enables them to bind target molecules with high specificity and affinity by leveraging van der Waals forces, hydrogen bonds, electrostatic interactions, and base stacking forces. Aptamers may potentially replace existing ligands and offer opportunities for improving treatment options for previously challenging diseases. A schematic representation of the aptamer application is illustrated in Figure 8.

- Dual role of aptamers: serving as modulators and therapeutic molecules

The strategy of utilizing aptamers as therapeutic antagonists is relatively straightforward and has been widely employed in ocular drug delivery systems [107]. The first aptamer drug, pegaptanib (Macaugen), was approved by the US Food and Drug Administration (FDA) in 2004 for treating age-related macular degeneration (AMD) [143]. Pegaptanib exhibits high affinity and specificity for the VEGF165 isoform, which is overexpressed in AMD and diabetic macular edema [63,144]. By preventing VEGF165 from binding to human umbilical vein endothelial cell receptors, pegaptanib can control neovascularization and, consequently, halt vision loss. Another example is E10030 (Fovista TM), an aptamer for AMD developed by Ophthotech Corp (New York, NY, USA). This aptamer binds to platelet-derived growth factor (PDGF) and inhibits its activity, thereby not only degenerating existing blood vessels, but also suppressing new blood vessel formation [145]. Though using aptamers as antagonists to directly block target molecule function is an attractive therapeutic approach, as it does not require cellular entry, there are considerable factors to take into account. Their practical application is limited due to the need for good binding affinity with target molecules and the consideration of potential side effects, such as metabolic instability and renal filtration failure. Hybrid nanocarriers combined with aptamers offer a promising approach to overcoming these limitations, as they can provide high penetration and therapeutic levels while improving these downsides.

- Aptamers as surface modifiers in drug carriers

Nanocarriers modified with aptamers hold great potential for treating a wide range of diseases, thanks to their ability to control functionality based on the medical target structure of aptamers. Additionally, the properties and therapeutic capabilities of these nanocarriers can be tailored depending on the drugs they carry [146,147]. For example, Lohiya G’s team demonstrated the possibility of targeted breast cancer treatment by modifying the surface of mesoporous silica nanoparticles (MSNs) with aptamers [148]. The developed aptamer-functionalized MSNs exhibited higher uptake and cytotoxicity in HER2-positive breast cancer cells when compared to non-targeted MSNs. This delivery system is expected to increase the therapeutic dose of the drug specifically utilized in cancer cells while minimizing the overall dose required to eradicate tumor cells.

**Figure 9:**
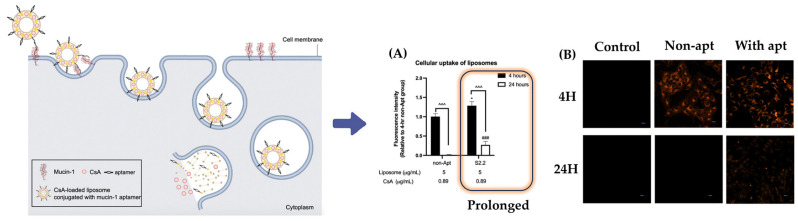
Study of nanocarrier-mediated drug delivery using aptamer interactions with mucin. (**A**) Verification of intracellular drug particle detection at 24 h in the presence of aptamers (quantification of fluorescence intensity). (* *p* < 0.001 compared with 4 h non-Apt group; ### *p* < 0.001 compared with 24 h non-Apt group; ^^^ *p* < 0.01 compared between different time points, non-Apt: non-aptamer.) (**B**) Fluorescent images of HCECs cultured with different liposomes for 4 h and 24 h. Reprinted from [149], © 2023 Royal Society of Chemistry Ltd. (Cambridge, UK). All rights reserved.

The significant potential of aptamer-modified nanodrug delivery systems for cancer treatment implies that similar systems could also show promise in targeted treatments for post-ocular diseases. Recent studies on mucin-targeting aptamer-functionalized liposomes for the delivery of cyclosporine A for dry eye syndrome support this possibility. In this research, Wong’s team assessed the cytotoxicity, anti-inflammatory effects, regulation of cell permeability, and retention time of liposomes in the corneal epithelial cells of dry eye patients [149]. Cyclosporine A loaded into liposomes demonstrated reduced toxic effects in human corneal epithelial cells (HCECs), regardless of the drug’s quantity within the liposomes. This implies that drug carrierization effectively decreases cytotoxicity. The researchers observed that mucin aptamer-functionalized liposomes remained within cells for up to 24 h, suggesting that aptamers can enhance drug retention time and cell permeability (Figure 9). Moreover, an in vivo study with a rat dry eye disease (DED) model was conducted to demonstrate the efficacy of aptamer-functionalized liposomes in restoring tear production and corneal integrity. Furthermore, other investigations on aptamer derivatives for the inhibition of retinal angiogenesis have also been undertaken by Moreira et al. [150]. However, research on whether aptamer derivatives can actually reach the retina through in vivo experiments is yet to be conducted, indicating that additional experiments are likely needed. Nevertheless, the results showcasing the potential of aptamer-targeted treatments for ocular diseases highlight the immense value of ongoing research on aptamer-functionalized nanocarriers in ocular drug delivery systems.

## 4. Exploring DNA Nanostructures as Innovative Vehicles for Ophthalmic Drug Delivery

Past and recent studies have developed a range of nanocarriers for ocular drug delivery. Each carrier incorporated a functionalization strategy designed to address the specific limitations inherent in drug delivery. By utilizing passive targeting strategies that improve the permeability and retention effect through controlling physical and chemical properties, such as the combined impact of surface charge and surface chemistry on the size and shape of the structure, the diffusion of nanocarriers can be improved. Furthermore, it has been shown that the use of targeting molecules such as proteins, aptamers, and peptides in active targeting can enhance sensitivity toward target cells. This also mitigates the reduction in permeability caused by ocular tissue cell screening. Consequently, this contributes to improving the efficiency of drug delivery. By capitalizing on these complementary strategies, researchers can develop effective ocular drug delivery systems that improve treatment outcomes for eye conditions.

**Figure 10:**
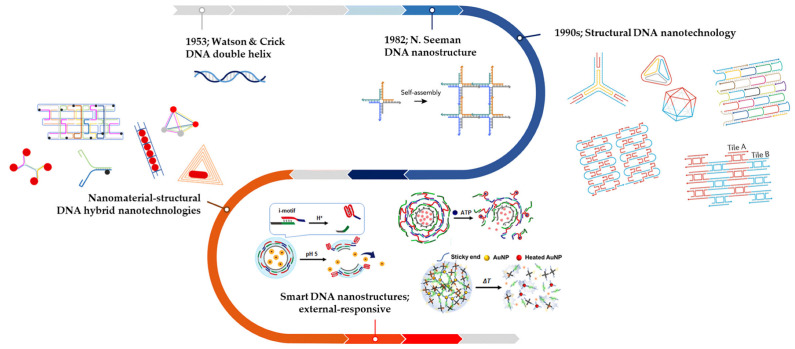
Schematic overview of the development process in structural DNA nanotechnology. This diagram illustrates the progression of DNA nanomaterials for practical applications, showcasing the diverse array of DNA nanostructures described in this review. This work is adapted with permission from: (1) [152] used under CC BY 4.0., (2) [153] © 2021 by Springer Nature (Berlin/Heidelberg, Germany), (3) [154] Copyright 2021 American Chemical Society (Washington, DC, USA), originally adapted from [155] Copyright 2016 American Chemical Society, [156] Copyright 2015 American Chemical Society, [157] © 2020 by John Wiley and Sons, Inc. (Hoboken, NJ, USA). All rights reserved.

DNA nanostructures, an emerging and rapidly advancing field in drug delivery research, present a next-generation active targeting platform that integrates proteins, aptamers, and peptides [151]. Traditionally, DNA has been considered a carrier of genetic information, but it is now also recognized as a smart material for the construction of nano-architectures in programmable and predictable patterns. Apart from its significance in genetic research and applications across various biological disciplines, such as biomedicine, cancer research, and genetic engineering, DNA’s unique properties—including structural stability, sequence programmability, and predictable self-assembly—have opened up new avenues in the realm of DNA nanostructures. Furthermore, stimulative DNA structures, such as those incorporating hydrogel systems, have shown promise in this field. Following extensive progress in structural design, DNA nanostructures have started to gain widespread application in the biomedical domain, heralding a new wave of disease treatment approaches. The development process of DNA-based nanostructures is presented in Figure 10.

DNA nanostructures possess a distinctive combination of advantageous properties, including biocompatibility, targeted delivery, design flexibility and precision, triggered release, adaptability, and compatibility with diverse drug types. These characteristics render DNA nanostructures a highly promising and attractive candidate for ocular drug delivery research and development. In this section, we delineate the design strategies employed in the construction of DNA-based nanocarriers and underscore their therapeutic successes in multiple domains. These accomplishments highlight the immense potential of DNA nanostructures as a viable approach for targeted drug delivery in the context of ocular diseases. By leveraging the unique attributes and self-assembly capacities of DNA nanostructures, we can devise innovative solutions to address challenges in ocular drug delivery and, ultimately, optimize the treatment of numerous eye-related disorders.

### 4.1. Early DNA Nanostructures

**Figure 11:**
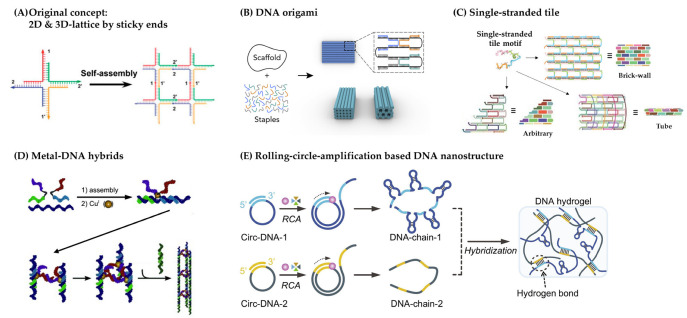
Schematic illustration of various methods for preparing DNA nanostructures. (**A**) Ned’s idea involves creating a 2D lattice from fixed junctions using complementary sticky ends. This DNA tile-based self-assembly, as exemplified by the assembly of a four-by-four DNA tile from various oligonucleotides, enables the construction of higher-order structures [159] used under CC BY 4.0., originally adapted from [158], Copyright 1982 Elsevier. (**B**) DNA origami transforms a long single-strand of DNA, or “scaffold”, into 2D or 3D structures using many short DNA oligonucleotides, known as “staples” [160], © 2021 Elsevier Ltd. All rights reserved. Originally adapted from [161], Copyright 2006 Springer Nature, and [162], Copyright 2011 Springer Nature. (**C**) Self-assembly of molecular shapes using single-stranded tiles [163], Copyright 2012 Springer Nature. (**D**) Metal-DNA hybrids represent a strategy to strengthen the binding capacity of nanostructures, achieved by integrating metal into the fundamental DNA assembly [164], © 2021 John Wiley and Sons. All rights reserved. (**E**) RCA, a process that enables the production of long single-stranded DNA using circular templates, is utilized in this context to generate extended DNA strands. These strands can then be assembled into desired nanostructures or further processed into a hydrogel, providing a versatile platform for various applications in nanotechnology and biomedicine [165], used under CC BY-NC-ND 4.0.

The discovery of DNA nanotechnology by Seeman in the early 1980s marked the beginning of the understanding of DNA as a potent material for building logically planned nanostructures outside the realm of biology. They invented DNA nanotechnology that showed the capability of generating nanocarriers of distinct sizes and geometries based on the major complementary base pairs of DNA, GC/AT [158]. Initially, the design of nanostructures was based on sticky binding between DNA strands, but it has progressed to offer several functionalities such as targeting by programming with other substances. Recently, researchers have developed smart DNA-based nanocarriers with dynamic DNA structures that can be switched reversibly [158]. These unique structures can initiate the release of encapsulated drugs into designated areas by reacting to specific stimuli such as strand displacement, pH alterations, and molecular or light-induced reconfigurations. This process effectively utilizes the high specificity and tunability inherent to DNA. Figure 11 provides a succinct overview of the fabrication process for these DNA nanostructures.

#### 4.1.1. Basic DNA Nanostructures: Polyhedron Assembly System

The structural design of DNA-based drug delivery nanocarriers, including 2D grids and 3D objects such as nanotubes, polyhedral, and other complex structures, for the application of DNA nano-systems begins with DNA self-assembly. The idea that DNA can be used as a nanoscale building material stems from the concept that “immovable junctions” can be assembled by rational designs of sequences and coupled to form 2D or 3D structures [166,167,168,169,170]. In fact, Seeman formed a 2D grid using complementary single-strand overhangs called sticky ends and constructed multiple shared-binding closed individual 3D objects such as cubes and tetrahedra based on terrain junctions [167,169,170,171]. Afterward, DNA tile-based self-assembly was proposed as a feasible approach to creating higher-order structures, including nanotubes and nanocages, using modular building blocks with adhesive end interactions [172,173,174].

The DNA origami strategy, first proposed by Rothemund, involves folding a long single-stranded DNA molecule (known as the “scaffold”) into a desired shape with the assistance of many short DNA oligonucleotides (known as “staples”) [161]. This concept has been expanded to design 3D origami structures by arranging DNA helices into different 3D lattices. This technique has progressed to enable the creation of complicated 3D DNA nanostructures with higher productivity and consistency, thereby simplifying the production of various 3D structures. By utilizing the principle of assigning the staple strands of DNA origami as unique pixels, it is possible to precisely fix other molecules such as functional biomolecules, ligands, and nano-sized objects in the desired positions, which offers the possibility of a versatile platform [161].

The development of DNA self-assembly has provided a way to simplify the design of complex DNA nanostructures. Single-stranded tile (SST) self-assembly allows DNA building blocks to form precise shapes without the need for scaffolds by creating loops with connected sticky ends [161]. SSTs interact with each other in a way similar to Lego bricks, through complementary domains. This approach demonstrates unlimited potential in geometric construction, capable of creating not only 2D patterns of complex structures, but also 3D shapes such as alphabets and other forms with complex surfaces, by assembling molecular canvases made up of numerous unique SSTs [163,175,176].

#### 4.1.2. Hybrid Nanostructures for Stability

In addition to DNA self-assembly, alternative strategies have been developed for constructing nanostructures, including the use of nanoparticle templates, metal DNA hybrids, and the rolling circle amplification (RCA) approach. Though single DNA duplexes have shown great potential, researchers have focused on enhancing the mechanical properties of DNA nanostructures.

In the early 1990s, Mirkin et al. achieved a significant breakthrough by creating the first hybrid structure combining nanoparticles and nucleic acids [177]. They employed gold nanoparticles as frameworks for the growth of short DNA strands. Terminal thiol-modified single-stranded DNA oligonucleotides attached to the surface of colloidal gold nanoparticles and duplexes (knowns as linkers) with complementary sticky ends on two transplanted sequences facilitated the self-assembly of gold nanoparticles into clusters. Mirkin observed that the distance between particles in the assembly was proportional to the length of the DNA linker [178,179]. This finding demonstrated that a robust core, combined with multiple double linkers, played a crucial role in maintaining the assembly of DNA-nanoparticle templates. This technique effectively increased the rigidity of the assembly. Subsequently, diverse inorganic nanoparticles, including catalytic noble metals, magnetic oxides, and semiconductors, were integrated with DNA. The DNA–nanoparticle superlattice, which allows for the formation of highly rigid DNA nanostructures through straightforward procedures, holds significant potential for expanding its applications in different types of domains.

The utilization of transition metals to facilitate the coupling and assembly of metal-DNA is another widely employed technique in the construction of DNA nanostructures, as mentioned earlier. This method involves creating DNA-metal hybrid nanostructures, where DNA double helices act as flexible appendages of rigid molecules on the nanoscale, centered around transition metals that can be precisely manipulated and controlled using specific organic molecule pockets [180]. In 2004, Sleiman et al. synthesized DNA complexes consisting of two parallel DNA strands linked to a Ru2-tris(bipyridine) core [181]. Through the design of DNA sequences that allow complementary hybridization, the self-assembly of ring-shaped metal-DNA nanostructures occurred. Subsequent research explored the use of copper ions for nanostructure formation [166]. By incorporating a copper-binding ligand, diphenyl phenanthroline, chemically synthesized between the two DNA strands, a highly stable complex that resisted denaturation during PAGE analysis was formed.

RCA (rolling circle amplification) has proven to be an effective method for efficiently creating DNA nanostructures. RCA involves amplifying multiple copies of a circular DNA template through a nucleic acid amplification technique [182]. These elongated DNA strands produced by RCA have been combined with origami folding techniques to construct a wide range of nanostructures, including nanoribbons, nanotubes, and nanospheres. In 2013, Ouyang et al. successfully developed an RCA-based nanoribbon using only 32 staple strands, demonstrating its easy internalization by cells [183]. Subsequently, with advancements in 3D topological structures based on DNA growth and paper folding, RCA has been employed as a means of merging or inserting therapeutic agents for applications such as drug delivery and targeted therapy [184]. One notable aspect of RCA is its ability to generate nanostructures without a predefined size, setting it apart from other DNA nanostructure formation methods. However, it is important to note that RCA-based nanostructures often have a high payload capacity due to their repetitive units, which should be considered when utilizing them.

### 4.2. Smart DNA Nanostructures in Therapeutic Drug Delivery

The self-assembly property of DNA has enabled the development of DNA nanostructures as highly attractive drug delivery carriers, particularly in terms of biocompatibility. Whereas conventional nanocarriers often face issues with biocompatibility and cell permeation due to their materials and size, DNA nanocarriers generally do not experience these challenges because of DNA’s inherent properties. However, controlling the drug release properties of DNA nanostructures can be challenging when compared to organic and inorganic nanocarriers, as DNA is sensitive to specific pH levels and oxidative environments, causing rapid decay [154]. Additionally, controlling the size and shape of the carrier using only the self-assembly process can pose difficulties. To address these challenges, researchers are attempting to stabilize DNA nanostructures with other materials, such as gold nanoparticles. Even so, this approach may not perfectly control drug release properties. To address this challenge, researchers are investigating the creation of intelligent drug delivery systems that integrate stimuli-responsive materials and DNA nanostructures. One strategy encompasses modifying the surface of DNA nanostructures by incorporating hydrogels, thereby enhancing the responsiveness of these nanocarriers to external stimuli. Smart DNA nanocarriers hold great promise, as they can potentially resolve the biocompatibility issues faced by the mentioned nanocarriers while also improving permeability due to in vivo interactions. Furthermore, these carriers can enable controlled drug release through external stimuli, such as pH changes, molecule interactions, and temperature fluctuations (Table 2). As research in this area continues to progress, the development of smart DNA nanocarriers is anticipated to contribute significantly to overcoming the limitations of current ocular drug delivery systems.

#### 4.2.1. Temperature-Responsive DNA Carrier

Temperature change has been used as a prominent external stimulus to trigger drug release from nanocarriers. The difference between external environment temperature and internal body temperature, as well as temperature changes due to inflammatory responses, enables the temperature to be an essential external stimulus, including the case of eyes [204,205]. The normal surface temperature of the eye is around 35 °C, and it can fluctuate up to 40 °C due to infection or other reasons. Specifically, the external temperature can act as an effective trigger for smart DNA nanocarriers, since double-stranded DNA unwinds into single-stranded DNA when hydrogen bonds between complementary base pairs break as the temperature increases. Researchers have made important advances leveraging temperature change as an external stimulus for DNA nanocarriers. Liu and colleagues developed a self-assembling DNA hydrogel exhibiting sensitivity to both temperature and enzyme reactions [206]. The Y-DNA and linker components formed a thermally sensitive pure DNA hydrogel through the complementary hybridization of sticky ends. However, this strategy does not adequately address temperature responsiveness and self-degradation in the human body, as the gel turns into a solution when temperatures rise from 25 °C to 50 °C due to the base pairing of complementary sticky ends. To overcome these limitations, researchers have incorporated nanomaterials with photothermal properties and nucleic acids to create DNA hydrogels that respond to temperature variations and degrade biologically. These hydrogels can be precisely controlled within the body. Notably, gold nanoparticles exhibit unique photothermal properties, allowing them to convert absorbed light energy into heat [188]. When integrated into a DNA hydrogel, these nanoparticles can act as an activator, initiating transitions between the gel and solution phases. Building on this concept, Song’s team designed a photothermally responsive, self-degradable DNA hydrogel embedding gold nanoparticles for rapid drug release in combined chemo-photothermal treatments [207]. As light illuminates the DOX-AuNP-DNA hydrogel, the resulting heat generation induces DNA hydrogel fragmentation and subsequent DOX release. Similarly, Cui’s group employed gold nanorods conjugated with interference oligonucleotides to develop an HA-DNA hydrogel aimed at gene therapy applications [187]. Here, near-infrared light increases the temperature, breaking the hydrogen bonds within the DNA helix and prompting the hydrogel’s degradation. This process releases the gold nanorods, which modulate pro-inflammatory genes. These instances showcase the potential for regulated drug delivery using DNA hydrogels.

#### 4.2.2. pH-Responsive DNA Carrier

Changes in pH are indeed one of the pathophysiological features that can be used to trigger drug release, especially in cancer therapies. Rapid cancer cell proliferation results in an acidic environment due to hydrolysis or protonation. As pH changes are also observed in the ocular environment in response to eye diseases, it can be considered an effective release control factor for DNA nanocarriers [208,209]. Researchers such as Zhang et al. have showcased pH-responsive metal-organic backbone DNA tetrahedral gates that release drugs in acidic environments due to the formation of quadruplexes through sequence reconstruction [210]. In another example, Song et al. achieved pH responsiveness in PEG-DNA-GNP carriers using an *i*-motif [190]. At normal physiological pH, drugs are stably incorporated into the M1/MC2 duplex. However, when the environment becomes slightly acidic, M1 forms an *i*-motif, causing MC2 to dissociate and release the drug. These studies demonstrate that pH-responsive DNA nanocarriers not only provide stable and high drug loading capacity, but they also enable control the release in response to intracellular endosomal/lysosomal acidic environments. This has the potential to improve the efficacy of drug delivery systems, particularly in ocular and cancer therapies, resulting in better clinical outcomes.

Alternative approaches to achieving pH responsiveness in DNA nanocarriers involve utilizing Watson–Crick- and Hoogsteen-type triplex motifs. These motifs form duplexes at neutral pH and they transition to TAT and CGC DNA triplets at lower pH values [211,212]. Yuwei et al. designed acid-resistant DNA hydrogels for stability in acidic environments by copolymerizing acrylamide monomers with adenine (A)- and cytosine (C)-rich oligonucleotides through free radical polymerization reactions [213]. Changes in Hoogsteen interactions and electrostatic forces, depending on the pH, induce binding or dissociation of the DNA hydrogel. In this study, pH-responsive DNA hydrogels were further developed for oral drug delivery against hostile acidic environments such as the stomach (pH 1.2), duodenum (pH 5.0), and small intestine (pH 7.2). Successful drug administration was confirmed through in vitro and in vivo studies. Moreover, Fu et al. sought to design a pH-responsive DNA motif that was not limited to a specific sequence [214]. By incorporating five pH-sensitive adenine/cytosine (A/C) mismatches evenly throughout the stem region, they successfully destabilized the hairpin structure, enabling it to lose its structure and hybridize with a 20-nucleotide-long DNA strand, forming a DNA duplex. However, when the pH changed during oxidation, the protonated adenine formed A/C base pairs with cytosine, causing the DNA duplex to dissociate. This dynamic process was shown to be reversible when the solution pH alternated between 5.0 and 8.0, highlighting its potential applicability in dynamic DNA nanotechnology.

#### 4.2.3. Biomarker Molecule-Responsive DNA Carrier

Small molecules found in the body, such as ATP and GSH, have the potential to act as endogenous stimuli that trigger conformational changes in DNA nanostructures. These small molecule-responsive DNA nanocarriers offer innovative perspectives for disease diagnosis and the development of therapeutic strategies. ATP concentrations vary in different intracellular and extracellular environments and among several organs in the body. Notably, intracellular and extracellular ATP levels are higher than normal in tumor cells or in the presence of inflammation [215]. This mechanism presents an appealing opportunity for designing DNA nanostructures that respond to ATP stimulation. Since DNA itself lacks ATP reactivity, most hybrid nano-assemblies are constructed using ATP aptamers [193,216,217]. Ran et al. introduced a strategy for drug delivery via nano-assemblies composed of graphene oxide, two single-stranded DNAs, and ATP aptamers [218]. Supramolecular π–π stacking interactions between graphene oxide and the drug resulted in high loading efficiency. It was confirmed that the formation of ATP/ATP aptamer complexes in the presence of ATP triggers the dissociation of nano-assemblies, promoting drug release in high ATP concentration environments, such as the cytosol, compared to ATP-deficient extracellular fluid. This approach paves the way for targeted drug delivery systems based on endogenous stimuli. In addition, Xu recently developed ATP-responsive DNA-polyacrylamide nanohydrogels using ATP aptamer as a cross-linker [219]. Following the gel–sol transition induced by ATP, DOX incorporated in the G-C bilayer structure was released, demonstrating anti-cancer cytotoxic effects. In the presence of high levels of intracellular ATP, the ATP aptamer competitively binds to ATP, preventing DNA strand hybridization and causing the disassembly of nanogel by grafting polyacrylamide backbone chains.

Glutathione (GSH), the most prevalent antioxidant molecule in organisms, serves as an excellent material for controlling the morphology of nanocarriers. Similar to ATP, GSH exists at levels four times higher in tumor tissue compared to normal cells and has a higher concentration within cells than in extracellular fluid. This makes GSH a viable candidate for efficient drug delivery in vivo [220]. In contrast to ATP-responsive nano-assemblies, which require ATP aptamers, GSH-responsive designs can employ disulfide cross-linked DNA, offering a significant advantage. Chen et al. reported the formation of DNA nanogels using cross-linking disulfide bonds that released drugs in response to elevated GSH levels [196]. Additionally, some researchers have constructed GSH-responsive nanocarriers through electrostatic interactions between materials and DNA. Specifically, Wang and colleagues developed polyplexes based on polymers containing p-(2,4-dinitrophenyloxybenzyl)-ammonium cationic moieties [221]. GSH specifically cleaves p-2,4-dinitrophenyl ether, converting the ammonium cation to a carboxylic acid anion. This charge-reversal mechanism allows for stable, GSH-responsive drug release while addressing the issue of conventional cationic polymers impeding intracellular release due to strong electrostatic binding to negatively charged DNA. Furthermore, research is broadening to include trigger-sensing drug delivery studies using different types of molecules such as specific enzymes, oligonucleotides, and metal ions [199,202,222,223,224]. It is anticipated that efforts will continue to develop methods that accurately release drugs within the complex environment of the human body.

### 4.3. Perspectives of DNA Nanocarriers for Ocular Drug Delivery

The ongoing research and development of DNA-based nanomaterials with varied structures and shapes for use in drug delivery systems highlight the promising potential of this field. Numerous studies have demonstrated that DNA nanocages can efficiently enter cells with the assistance of a transfection agent, such as lipofectamine, and can accumulate in the cytoplasm while maintaining their intact structure for up to 48 h [225]. Moreover, DNA nanotubes have exhibited no toxicity to living cells, even when combined with other molecules such as ligands or fluorescent dyes [226,227]. Despite continued efforts to utilize proteins or similar peptides found in the body to create drug delivery carriers with excellent biocompatibility, it has been reported that intraocular administration of nanoparticles modified with these materials can cause eye inflammation [228]. This underscores the importance of good biocompatibility when using DNA-based materials for ocular drug delivery [229]. Though most studies on DNA nanocarriers have focused on their preparation and application for cancer treatment, recent research has begun exploring their potential in ocular drug delivery systems (Table 3).

**Figure 12:**
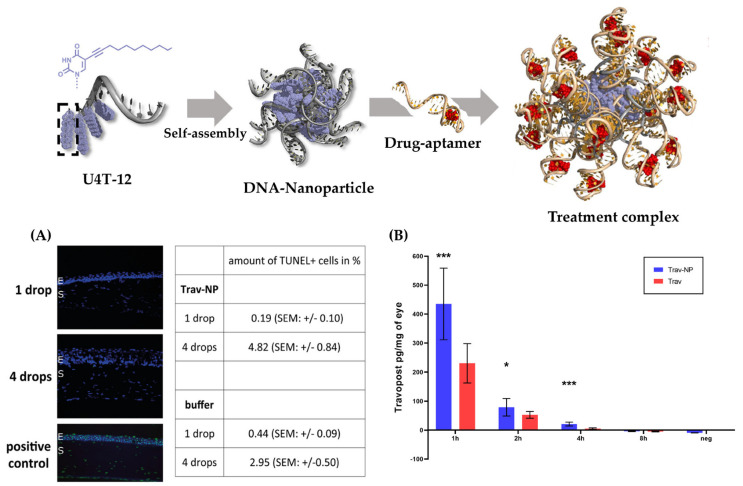
Characterization and evaluation of lipid DNA nanoparticles loaded with anti-glaucoma drug travoprost (Trav), facilitated by the hybridization of specific aptamers that bind to the drug. (**A**) Assessment of corneal apoptosis induction by NPs using TUNEL assay, revealing minimal apoptotic cells and confirming NP stability (rat corneas shown in blue and apoptotic cells shown in green; total average of TUNEL-positive epithelial cells per given epithelial cell expressed as % by SEM). E represents Epithelium while S denotes Stroma. (**B**) Enhanced uptake of travoprost by lipid-DNA NPs in vivo (statistical differences for raw travoprost indicated by * for *p* < 0.05 and *** for *p* < 0.001). This work is adapted with permission from [235], © 2020 Elsevier Ltd. All rights reserved.

Researchers such as Kim et al. used a single plasmid DNA molecule compressed with polyethylene glycol-substituted polylysine (CK30PEG) to deliver cystic fibrosis transmembrane modulators to patients with cystic fibrosis [240]. These DNA nanoparticles were introduced into retinal tissue using subretinal injections and did not cause any local toxicity or inflammation. Moreover, recent studies have shown that DNA nanoparticles are effective in eye drop delivery as well. Willem et al. developed drug delivery carriers by hybridizing oligonucleotides to DNA nanoparticles that surround a lipid core [231]. The experiment confirmed improved residence time in porcine and human corneal tissue compared to the original drug, highlighting the potential to increase the in vivo efficiency of these drug delivery systems. Subsequent studies conducted by other research groups have supported the potential of DNA nanocarriers for intraocular drug delivery [232,233,235]. In an in vivo experiment reported by one research group, nanoparticles were injected into the eye tissue of rats and it was found that although the drug appeared diffused when compared to injections into the vitreous cavity, adhesion to retinal tissue was still achieved up to 5 days [232]. As demonstrated in another study, the nanoparticles effectively lowered intraocular pressure compared to the native drug due to the increased adhesion of NPs to the corneal surface for up to 4 h in vitro and up to 1 h in vivo (in pig eyes and rats) [233]. In a separate investigation, Trav-NP was shown to maintain the drug effect for up to 4 h following eye drop instillation and outstanding biocompatibility was confirmed without any indications of apoptosis (Figure 12) [235].

**Figure 13:**
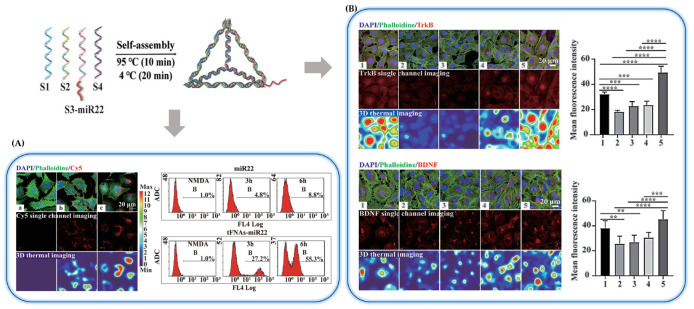
The potential of DNA nanocomposites synthesized by attaching miR-22 to DNA carriers (tFNA) for the treatment of optic nerve degenerative diseases. (**A**) Enhanced uptake of miR-22 in RGC-5 cells treated with NMDA due to tFNA (Cy5-miR22 and Cy5-tFNA-miR22: red; nucleus: blue; cytoskeleton: green); (**a**) NMDA; (**b**) miR-22-Cy5; (**c**) tFNAs-miR22-Cy5. (**B**) Selective activation of TrkB and restoration of BDNF in damaged retinal neurons by tFNA-miR22; expression and quantitative analysis of TrkB in RGC-5 using immunofluorescence confocal microscopy (cytoskeleton: green, nucleus: blue, TrkB: red); and expression and quantitative analysis of BDNF in RGC-5 using immunofluorescence confocal microscopy (cytoskeleton: green, nucleus: blue, BDNF: red). Statistical analysis: ** *p* < 0.01, *** *p* < 0.001, **** *p* < 0.0001. GAPDH was employed as an internal control; (1) Control; (2) NMDA; (3) NMDA + miR-22; (4) NMDA + tFNAs; (5) NMDA + tFNAs-miR22. This work is adapted with permission from [237], © 2021 by John Wiley & Sons, Inc. All rights reserved.

In a recently published study on ocular drug delivery involving DNA nanocarriers, researchers developed a DNA nanocomposite by using microRNA instead of organic or inorganic materials [237]. In this study, they selected tetrahedral frame nucleic acid (tFNA), which has biological functions, as the carrier. They attached microRNA-22-3p (miR-22) to the tFNA to treat progressive retinal ganglion cell loss and axonal damage caused by glaucoma. The study demonstrated that tFNA could facilitate miR-22 uptake in retinal neurons, as evident from the results in NMDA-treated RGC-5 cells (Figure 13A). NMDA-treated RGC-5 cells were divided into time-based groups and exposed to Cy5-miR-22 and Cy5-tFNA-miR22, respectively. Notably, the Cy5 fluorescence of tFNA-miR22 started increasing significantly at 3 h and peaked at 6 h, whereas the Cy5 fluorescence of miR-22 remained faint at this time point. Although miR-22 gradually entered cells over 24 h, its entry efficiency at 24 h was still lower than that of tFNA-miR22 (33.5% vs. 40.4%). In subsequent experiments aimed at assessing the ability of tFNA-miR22 to modulate the TrkB-BDNF signaling pathway in retinal neurons, tFNA-miR22 demonstrated greater efficacy compared to other groups (Figure 13B). Consistent with expectations, both mRNA and protein levels of BDNF were significantly reduced after NMDA induction in the study. However, after treatment with tFNAs-miR22, both TrkB and BDNF expression levels were significantly higher compared to other groups. These findings indicate that tFNAs-miR22 selectively activates TrkB and restores BDNF expression in damaged retinal neurons. These findings not only demonstrate the potential of DNA nanostructures in the treatment of eye diseases, but also highlight the successful establishment of a straightforward, yet effective, delivery system for relevant microRNAs.

Though the present paper did not delve extensively into the topic of microRNA delivery, ongoing research in the drug delivery field aims to treat diseases by directly delivering microRNA, akin to the use of aptamers as drug molecules. In particular, since the emergence of COVID-19, interest in nucleic acid therapeutics has surged, with diverse applications being explored, including the treatment of eye diseases. In neovascular eye disorders such as diabetic retinopathy, age-related macular degeneration, and retinopathy of prematurity, miRNA expression levels are known to be disrupted [241]. As such, delivering miRNA not only holds promise for treating ganglion cell damage caused by glaucoma, but also suggests the potential for addressing neovascularization induced by a variety of diseases. Given that mRNA is a type of nucleic acid, it is highly unstable, susceptible to rapid degradation due to environmental changes, specific proteins, and being recognized as an endogenous molecule, potentially triggering immune responses. Consequently, a carrier is necessary. Lipid nanoparticles (LNPs) have been widely employed as carriers for mRNA. However, when utilizing DNA nanostructures, it is possible to control interactions with mRNA by diversifying the structure and properties of the materials, thus underscoring the significance of DNA nanostructures in mRNA delivery in future developments. Moreover, previous studies have demonstrated the efficacy of combining DNA nanostructures and mRNA in treating ocular conditions. As a result, research in this area is expected to continue gaining attention and contribute to advancements in targeted therapies for eye diseases.

Indeed, the progression of research on ocular drug delivery carriers has frequently seen carriers that demonstrate effectiveness in systemic diseases such as cancer extend their applicability to the realm of ocular treatment. For instance, Viral S. Kansara et al.’s study examined the therapeutic effect of DNA nanoparticles (DNPs) consisting of single DNA molecules compacted with 10 kDa polyethylene glycol (PEG)-substituted lysine 30-mers (CK30PEG) in retinal diseases [238]. Prior to investigating the treatment impact on retinal disorders, the researchers conducted a study on cystic fibrosis. It is clear that ocular drug delivery utilizing several DNA nanocarriers, such as the previously described hydrogel-based smart DNA nanocarrier, has not yet been extensively explored. However, considering that hydrogel-based ocular drug delivery systems are evaluated as promising and extensively used in ocular drug delivery and that carriers proven effective in other fields have extended to the realm of ocular treatment, hydrogel-based smart DNA nanocarriers are indispensable for the future of ocular drug delivery. Moreover, due to the success of integrating DNA nanocarriers with mRNA in treating ocular diseases and the increasing interest and progress in the field of nucleic acid therapeutics, there is substantial potential for further development and innovation in ocular drug delivery using DNA nanocarriers.

### 4.4. Challenges in Utilizing DNA Nanocarriers for Ocular Drug Delivery

DNA nanostructures hold promise in the field of ocular drug delivery; however, they currently face a myriad of challenges and opportunities. Practical application difficulties stem from variations in drug circulation, distribution, metabolism, potency, and degradation, which depend on the interactions between DNA and cellular behavior. The potential risks associated with DNA nanostructures for ocular drug delivery parallel those observed in nanostructures designed to address toxicity, side effects, and drug resistance challenges in anti-tumor therapies [183,242]. As a deoxynucleotide polymer, DNA is prone to degradation in blood circulation. Furthermore, due to its small size and biocompatibility, DNA can readily bind to single-stranded RNA, such as genes in the cell nucleus, which may result in dysregulation of gene expression. Despite these risks, the high biocompatibility of DNA, a naturally occurring biological molecule, underscores its potential for further development into complex and advanced forms to achieve efficient medical applications. Presently, this field remains in its early stages and practical research focused on biomedical applications is limited. Predominantly, studies are conducted on in vitro cell cultures, tissues, or animal models, such as mice and rabbits. To expand the use of DNA nanostructure-based drug delivery systems in human medicine, researchers must consider immune and circulatory functions, as well as address the challenges of high-purity manufacturing and mass production of DNA nanostructures, which are factors that significantly impact commercial applications. Rigorous preclinical and clinical trials, combined with innovative manufacturing techniques, will be instrumental in overcoming the current limitations and bringing these novel drug delivery systems closer to clinical application. Ultimately, these efforts may lead to groundbreaking therapies for ocular diseases, improving eye care and patients’ quality of life.

## 5. Conclusions

In conclusion, the field of ocular drug delivery is progressing rapidly, with novel technologies and strategies being developed continuously to address the unique anatomy and physiology of the eye. This current review highlights and summarizes pertinent outcomes of attempts to overcome barriers in ocular drug delivery systems including hydrogel-based carriers and presents the efforts and advancements aimed at enhancing targeting for future ocular drug therapies. Among these, nanomaterials are playing a significant role in transforming the landscape of ocular drug delivery systems. Drug delivery carriers based on nanomaterials hold remarkable clinical translation potential for the treatment of a broad range of eye diseases. These diseases encompass numerous conditions, such as glaucoma, age-related macular degeneration, diabetic retinopathy, and several types of ocular inflammation. Given the diverse applications of nanocarriers, they may even replace conventional eye drops in the near future. Molecules such as proteins, peptides, and aptamers demonstrate excellent targeting effects, indicating their combination with nanocarriers that can treat diseases together can substantially aid in addressing the challenges of ocular drug delivery. This amalgamation of molecular targeting and nanocarriers has the potential to revolutionize the way ocular diseases are managed and treated, ultimately leading to better patient health outcomes. Furthermore, considering the advantages of DNA nanostructures, such as biocompatibility, targeted delivery, design flexibility and precision, induced release, adaptability, and compatibility with multiple drug types, it is no surprise that there is a growing interest in DNA nanostructures within the field of drug therapy. DNA nanostructures are particularly appealing because they are versatile platforms for the delivery of diverse therapeutic agents. As applied research shifts from constructing DNA nanostructures toward practical applications, their promise as an innovative approach for addressing ocular diseases grows increasingly evident. Researchers persist in investigating techniques for optimizing these structures for efficient drug delivery, increased tissue penetration, and minimized potential toxicity. The field is rich with opportunities for innovation, as scientists work tirelessly to develop new carriers and functionalization strategies that can circumvent the many challenges in delivering medications to the eye. In summary, the rapid advancement of ocular drug delivery technologies, combined with the development of targeted nanocarriers such as protein peptides, aptamers, and promising DNA nanostructures, signals a bright future for ocular therapeutics. These advancements are anticipated to lead to substantially improved treatment options for patients suffering from a wide array of eye diseases. As the comprehension of ocular drug delivery progresses and the field develops, significant breakthroughs can be anticipated in the forthcoming years, culminating in improved treatment outcomes and enriched quality of life for individuals challenged by eye conditions.

## Figures and Tables

**Table 2:** Smart DNA nanostructures for stimuli-responsive drug delivery.

Stimulus		Structures	Mechanisms	Target Diseases (Drug)	Ref.
Temperature		DNA based silver nanoclusters	The anti-parallel four-strand structure forming DNA-AgNC is structured as an *i*-motif including C-quadruplex as the temperature changes.	Cancer (Dox)	[185]
		DNA-gated mesoporous silica nanocarriers	Change of the amino group on the surface of the MSNs acting as the valve	Cancer (Dox)	[186]
		DNA-grafted HA with gold nanorod	NIR-triggered on-demand release of spherical nucleic acids by photo-thermal induced DNA dehybridization	Osteoarthritis (gene therapy)	[187]
		DNA based hydrogels loaded with gold or silver nanoparticles	Thermoplastic properties of AuNPs and AuNRs trigger the dehybridization of the DNA duplexes	Cancer (Dox)	[188]
pH		Mg^2+^ aggregated functional DNAs from RCA (*i*-motif)	*i*-motif structure switch in response to pH changes	Cancer (Dox)	[189]
		MN/MC2 duplex with GNP (*i*-motif)		Cancer (Dox)	[190]
		DNA polymer micelles (Hoogsteen-type triplexes)	Hoogsteen interaction switch in response to pH changes	Cancer (Dox)	[191]
		Tetrameric DNA walker (triple-stranded structure)		- (Fluorescence)	[192]
Biomolecule	ATP	Framework nucleic acid (FNA) nanocarriers	ATP aptamer (ABA27) responding to ATP triggers the toehold-mediated strand displacement reaction	- (mRNA)	[193]
		2D MoS_2_ Nanosheets with DNA	Autonomously disassembled of structures in response to cancer cells’ heightened ATP metabolism	Cancer (Dox)	[194]
		DNA hydrogels by aptamer-trigger-clamped hybridization chain reaction	Destruction of the hydrogel through the stimulus-response of ATP	Cancer (cloaking and decloaking of tumor cells)	[195]
	GSH	DNA-DOX nanogels formed by Cross-linking kiwifruit-derived DNA	High GSH concentration cleaved the disulfide bonds of DTSSP-cross-linked DNA-DOX NGs	Cancer (Dox)	[196]
		DNA nanohydrogels were created through a self-assembly process using three kinds of building units	High GSH concentration cleaved the disulfide bonds of building units (Y-shaped monomers and a DNA linker)	Cancer (-)	[197]
		DNA nanodevice functionalized with small interfering RNA (siRNA)	Mechanical opening and release of siRNA in response to intracellular GSH; cleaved the disulfide bonds	Cancer (Dox)	[198]
	Enzymes	Artificial kinase-mediated cascade nanosystem composed of nanomediator (NM) and nanoeffector (NE)	Protein kinase-catalyzed phosphorylation to secondary mediator DNA	Cancer (Dox)	[199]
		Nanocarriers with double-stranded DNA and MMP-2 cleavable peptides	(MMP)-2 enzymes overexpressed in tumor tissue cleaved the peptide chain	Lung cancer (Dox)	[200]
	Oligonucleotides	Spherical nucleic acid from monodisperse DNA–polymer conjugates	In the present of a particular cytoplasmic genetic marker, two triggers hybridize and release nucleic acid therapeutics.	- (Nucleic acid therapeutics)	[201]
		Drug delivery platform of carbon dots which were connected to a stem-loop molecular beacon	Overexpressed endogenous microRNA-21 released drugs by competitive hybridization with the molecular beacon	Cancer (Dox)	[202]
	Metal ion	Loop size of the DNA hairpin	Formation of Thymine–Hg(II)–Thymine complexes by DNA–Hg(II) interactions	- (detection of mercury(II))	[203]

**Table 3:** DNA nanocarriers for ocular drug delivery.

Structures	Effectiveness	Target Diseases	Ref.
DNA nanoparticles	Excellent biosafety profile in-vitro Offering the opportunity to deliver hydrophobic and hydrophilic drugs	Virus or infections	[230,231]
Lipid-DNA Nanoparticles	Long-lasting adherence Excellent biosafety profile in-vitro or in-vivo Increased therapeutic effect	Retinal diseases or glaucoma	[232,233,234,235]
Tetrahedral framework nucleic acids	Enhanced endocytosis Promoting the normalization of disrupted vasculature	Optic neurodegenerative diseases (gene delivery)	[236,237]
Plasmid DNA nanoparticles compacted with PEG-substituted lysine 30-mer peptides	Well tolerated, with no significant ocular examination score changes Higher delivery to the retinal space	Retinal diseases	[238,239]

## Data Availability

Not applicable.
